# Sensitive and selective determination of imidacloprid with magnetic molecularly imprinted polymer by using LC/Q-TOF/MS

**DOI:** 10.3906/kim-2101-36

**Published:** 2021-08-27

**Authors:** Raif İLKTAÇ, Zinar Pınar GÜMÜŞ

**Affiliations:** 1 Central Research Testing and Analysis Laboratory Research and Application Center, Ege University Bornova, İzmir Turkey

**Keywords:** Imidacloprid, high resolution chromatography, molecularly imprinted polymer

## Abstract

In this paper, magnetic-molecularly imprinted polymer was used for the preconcentration of trace levels of imidacloprid in water and apple samples prior to liquid chromatography-quadrupole-time-of-flight mass spectrometric determination. The selectivity of the magnetic polymer was united with the sensitivity and the high resolving power of the chromatographic system. The developed method showed a linear range from 10.0 to 500.0 µg/L. The quantitative recoveries were obtained for water and apple samples in the range of 92.0%–99.0 %. The relative standard deviations of intra-day and inter-day tests were found to be in the range of 0.8%–1.2% and 1.2%–1.6 %, respectively. In addition, the same magnetic-molecularly imprinted polymer (MMIP) can be used at least ten cycles for the determination of imidacloprid. The preconcentration factor of the method was found to be 2.5, and the total preconcentration procedure can be completed in 1 h. Characterization of synthesised particles were executed with various techniques. Due to its suitable limit of detection, dynamic linear range, sensitivity and selectivity, the developed method seemed to be ideal for the determination and preconcentration of imidacloprid in water and fruit samples.

## 1. Introduction

Imidacloprid [1-(6-chloro-3-pyridylmethyl)-N- nitroimidazolodin-2-ylideneamine] is a systemic neonicotinoid insecticide, which acts as an insect neurotoxin on the central nervous system of insects [1]. Environmental samples may be contaminated due to their permanence in tissues and even very small concentrations of neonicotinoids may cause carcinogenic and mutagenic effects [2]. Imidacloprid has been widely used in various types of fruits and vegetables in agriculture and also in forestry. Upon its high persistence, imidacloprid is absorbed by plant roots and can be transported through all plant organelles [3,4]. As a result of its widespread usage in agricultural applications, the residue of imidacloprid in ground and/or surface waters, soil and food products become an important hazard to human health [5–8]. Thus, the effect of imidacloprid on food and aquatic environment is more important today than ever before. 

Determination of trace levels of imidacloprid is quite important since the European Union (EU) set the maximum residue limit (MRL) value for imidacloprid as 0.5 mg/kg for apple [9]. Chromatographic techniques are the most commonly used techniques in pesticide analysis [10–12]. Sample preparation and preconcentration are of great importance for analysis of trace levels of pesticides, since the sensitivity and selectivity of the methods depend on sample preparation and cleaning prior to analysis [13].

Solid phase extraction (SPE) [14–16], solid phase microextraction (SPME) [17–19], liquid-liquid extraction (LLE)[20,21], dispersive micro-solid phase extraction (d-SPME) [22,23] magnetic solid-phase extraction [24,25], dispersive liquid-liquid microextraction (d - LLE) [26,27] and ultrasonic extraction (UE) [28] techniques have been widely used in sample preparation step prior to chromatographic pesticide detection. However, methods based on more selective sorbents with the suitable recovery and enrichment abilities need to be developed especially in complex sample matrices [29,30].

Beside these techniques, molecularly imprinted polymers (MIPs) are also used in the analysis of the pesticides. MIPs can be defined as commonly artificial molecular recognition materials, obtained by the polymerization reaction of a functional monomer with a template. Upon the removal of the template (analyte) after polymerization, specific binding sites are produced in the polymer structure, and MIPs can be used for the selective sorption of the analyte via the specific binding sites, geometry and functionality [31].

MIPs have many important properties, such as having simple and low-cost synthesis, high chemical stability and mechanical strength, strong recognition ability, high selectivity, reusability and reproducibility [32–34]. Therefore, MIPs play an active role in the separation, determination, preconcentration of pesticides and elimination of interferences in environmental and food samples [35]. MIPs also possess some drawbacks such as slow reaction rates and effortful separation of polymer from the solution [36,37].

As a fast and effective technology, magnetic separation technology has been combined with traditional MIP magnetic technology in recent years to prepare magnetic-molecularly imprinted polymers (MMIPs). In MMIPs, the advantages of MIP are preserved, and at the same time, they possess high specific surface area and excellent magnetic properties, which can increase the efficiency and eliminate the time consuming step of separating the polymer from the complex sample using an external magnet [38,39]. Determination of imidacloprid based on different types of MIPs with various techniques and different components were presented in the literature [40–42]. In this study, MMIPs have been used for the preconcentration of trace levels of imidacloprid prior to the chromatographic separation and highly sensitive mass spectrometric detection. MMIPs not only preconcentrate the analyte but also eliminate the matrix effects and minimize the ion source contamination. Thus, the MMIP based chromatographic method is expected to set a different perspective for the analysis of trace levels of imidacloprid in a complex sample matrices.

The aim of the study is to unite the selectiveness of the magnetic polymer with the sensitivity and the high resolution of the chromatographic system for determining imidacloprid. The method was applied to tap water and apple samples, and the results demonstrated that the developed method for recovery of imidacloprid was a promising tool and will shed light on methodological evaluations for the detection of imidacloprid in real samples. 

## 2. Experimental

### 2.1. Reagents

Imidacloprid, thiabendazole (98 %), methacrylic acid, acetamiprid, chloroform, ethanol, carbofuran (98 %), acetonitrile, 2,2¢-azobis(isobutyronitrile), pirimicarb, carbendazim (97 %), ethylene glycol dimethacrylate (97 %), FeCl_2_.4H_2_O, FeCl_3_.6H_2_O and methanol were purchased from Merck (St. Louis, MO, USA). Thifensulfuron-methyl (97 %), chlorothalonil (98 %), tebuconazole (98 %) and triclosan (> 98 %) were purchased from TCI (Portland, OR, USA). Thiram was obtained from Alfa Aesar (Haverhill, MA, USA). Ultrapure (type 1) water was used in the studies (Millipore, Bedford, MA, USA). 1000 µg/mL stock imidacloprid solution was prepared in methanol.

### 2.1. Apparatus

The Agilent 1260 Infinity LC system coupled to 6550 iFunnel high resolution accurate-mass quadrupole-time of flight mass spectrometer was used for the separation, identification and quantification of imidacloprid. The high-resolution MS system was operated with an Agilent Dual Jet Stream electrospray in positive ionization mode (Agilent Technologies, Santa Clara, CA, USA). 

Fourier transform infrared analysis was executed by Spectrum Two FTIR spectrometer (Perkin-Elmer, Norwalk, CT, USA). The morphological and elemental analysis of the synthesized particles were identified by scanning electron microscope-energy dispersive X-ray spectroscopy (SEM-EDX) (Thermo Scientific, Waltham, MA, USA). Magnetic properties of magnetite particles and magnetic-molecularly imprinted polymer were determined with vibrating sample magnetometer (VSM) (Lakeshore, Westerville, OH, USA).

### 2.2. Preparation of magnetite (Fe3O4) particles

Co-precipitation method was used for the synthesis of magnetite particles [43]. FeCl_2_.4H_2_O (0.01 mol) and FeCl_3_.6H_2_O (0.02 mol) were dissolved in 100 mL ultrapure water, and the solution was heated up to 80 °C while purging with N_2_ gas. Upon the addition of 50.0 mL 2.0 M NaOH solution, magnetite particles were obtained as black precipitates. After one hour, magnetite particles were separated from the solution with a magnet and washed with ultrapure water.

### 2.3. Preparation of magnetic-molecularly imprinted polymer (MMIP)

Magnetic-molecularly imprinted polymers were prepared with the slightly modified protocol (absence of the ultrasound) as mentioned in the literature [44] : 0.5 mmol imidacloprid was added with 2.0 mmol functional monomer, methacrylic acid, in 5.0 mL acetonitrile. Methacrylic acid was chosen since it has the ability to form hydrogen bonds with template, and acetonitrile was chosen since it is a polar aprotic solvent. After stirring for 30 min, 10.0 mmol cross-linker, ethylene glycol dimethacrylate, was included into the system. Elsewhere, 5.0 mL oleic acid was stirred with 0.5 g magnetite particles for 10 min. Then, these two solutions were mixed.

Saturated polyvinyl alcohol (PVA) solution, the dispersing agent, was added to the mixture, and the mixture was homogenized with a mechanical stirrer under N_2_ atmosphere. In the last step, 100 mg 2,2¢-azobis(isobutyronitrile), the initiator, was added, and the reaction was allowed to be proceeded at 60 °C under N_2_ atmosphere for 4 h. Then, synthesized MMIP was separated from the mixture with a magnet.

Imidacloprid was extracted from the MMIP structure by washing with methanol : acetic acid (9 : 1) and methanol until imidacloprid peak could not be observed by the high resolution MS system. Same procedure was followed for the synthesis of magnetite non-imprinted polymer (MNIP) without using imidacloprid. 

### 2.4. Re-binding and recovery studies for imidacloprid detection

In order to re-bind imidacloprid, 100 mg polymer (MMIP/MNIP) was shaken with 5.0 mL, 0.5 µg/mLimidacloprid solution for 30 min at room temperature. After sorption, magnetic separation was established, and the solution was injected to LC/Q-TOF/MS. 

For recovery of imidacloprid, in the first step, re-binding procedure was followed, and upon sorption, MMIP was separated from the solution. 5.0 mL methanol was added onto MMIP, and the mixture was shaken for 30 min to recover the pesticide. Imidacloprid was determined by LC/Q-TOF/MS.

The instrumental conditions for chromatographic separation and determination of imidacloprid were given in Supplemental Material. LC/Q-TOF/MS extract ion chromatogram (EIC) and Pesticides Accurate Mass Personal Compound Database Library (Pesticides_AM_PCDL) identification of imidacloprid were illustrated in Figure S1. 

## 3. Results and discussion

3.1. Characterization studies

Characterization of MMIPs were performed with FTIR, SEM-EDX and VSM analysis.

FTIR spectra of magnetic particles and MMIP can be shown in Figure S2. The peaks at 2930 cm^–1^, 1720 cm^–1^ and 1140 cm^–1^ were the proofs of a successful polymerization associating with C-H stretching, C=O stretching and C-O stretching, respectively.

**Figure 1:**
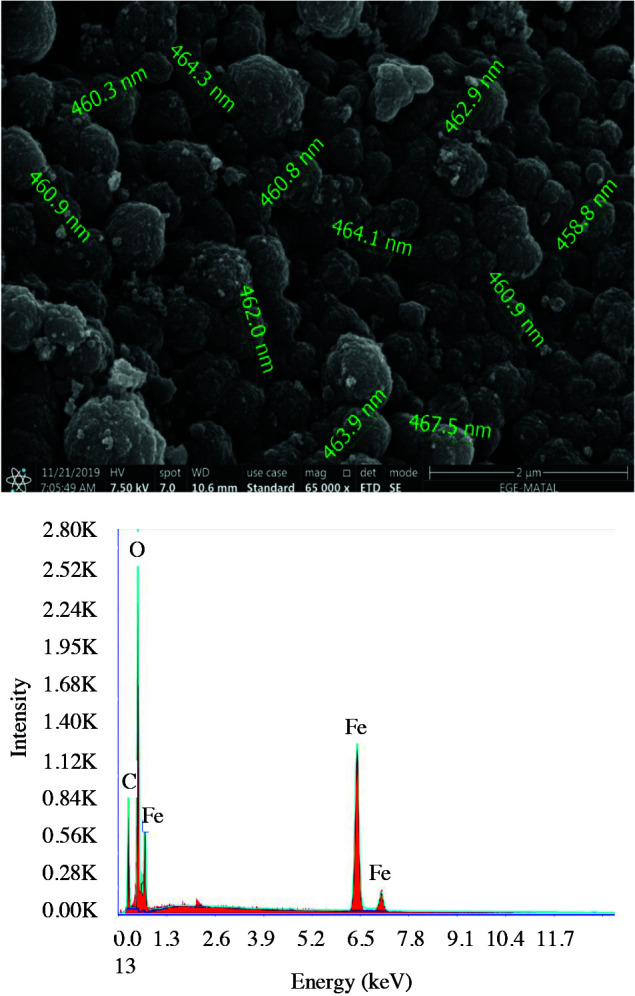
SEM-EDX analysis of MMIP.

As can be seen from the SEM image in Figure 1, particles consisted of a spherical and porous surface with the average diameter of 462.4 nm. According to EDX results, iron (Fe) and oxygen (O) and carbon (C) were detected in the surface of MMIP. Existence of carbon in MMIP verified the polymer structure on the surface of magnetite.

Figure S3 illustrates the VSM analysis of magnetic particles and MMIP. According to Figure S3, the saturation magnetization value (M_S_) of MMIP (12.0 emu/g) was smaller than the M_S_ value of magnetic particles (52.7 emu/g). Therefore, it can be concluded that the saturation magnetization reduced after the formation of the polymer layer on the surface of the magnetic particles.

### 3.1. Solvent choice on re-binding and recovery of imidacloprid

Both the re-binding efficiency and recovery of imidacloprid were investigated using different solvents such as water, methanol : water (20 : 80, v/v), ethanol, acetonitrile, methanol : water (50 : 50, v/v), methanol : water (80 : 20, v/v), chloroform and methanol. The main reasons for investigating the effects of methanol, ethanol and water on re-binding efficiency and recovery are as follows: i) their ability to form hydrogen bonds, ii) they are the most widely used solvents in the MIP studies. The effects of acetonitrile and chloroform were also investigated, since they are the mostly used porogens in imprinting process. Table 1 shows the effects of solvents on re-binding and recovery of imidacloprid.

**Table 1:** Effect of solvents on re-binding and recovery of imidacloprid.

Sample	Solvent	Re-binding Efficiency (%)	Recovery (%)*
	acetonitrile	Not significant	39.6 ± 2.1 %
	chloroform	13.3 ± 6.1 %	32.9 ± 7.2 %
	ethanol	35.4 ± 4.5 %	44.3 ± 4.1 %
	methanol	17.4 ± 2.9 %	97.0 ± 2.5 %
	water, pH ~ 4	51.3 ± 2.6 %	Not significant
	water, pH ~ 7	90.5 ± 2.4 %	Not significant
MMIP	water, pH ~ 9	84.5 ± 3.9 %	Not significant
	methanol : water (20 : 80, v/v), pH ~ 7	88.5 ± 2.4 %	Not significant
	methanol : water (50 : 50, v/v), pH ~ 4	56.3 ± 4.0 %	Not significant
	methanol : water (50 : 50, v/v), pH ~ 7	96.3 ± 1.5 %	Not significant
	methanol : water (50 : 50, v/v), pH ~ 9	90.7 ± 2.5 %	Not significant
	methanol : water (80 : 20, v/v), pH ~ 7	81.1 ± 4.7 %	Not significant
	water, pH ~ 4	43.9 ± 3.5 %	
	water, pH ~ 7	62.2 ± 5.4 %	
	water, pH ~ 9	55.5 ± 4.1 %	
	methanol : water (20 : 80, v/v), pH ~ 7	53.1 ± 3.5 %	
MNIP	methanol : water (50 : 50, v/v), pH ~ 4	40.6 ± 3.1 %	
	methanol : water (50 : 50, v/v), pH ~ 7	42.7 ± 3.5 %	
	methanol : water (50 : 50, v/v), pH ~ 9	46.6 ± 3.9 %	
	methanol : water (80 : 20, v/v), pH ~ 7	43.3 ± 3.5 %	

In the re-binding studies, it was found that, in methanol : water (50 : 50, v/v, pH ~ 7), MMIP could re-bind imidacloprid quantitatively, 96.3 ± 1.5 % (n = 3), whereas imidacloprid re-binding efficiency onto MNIP was 42.7 ± 3.5 % (n = 3).

The pH effect of the solution was investigated for methanol : water (50 : 50, v/v) medium for pH values of 4, 7 and 9 by using buffer solutions. It was found that the re-binding efficiencies of imidacloprid onto MMIP were found to be 56.3 ± 4.0 %, 96.3 ± 1.5 % and 90.7 ± 2.5 % (n = 3), for pH values of 4, 7 and 9, respectively. However, for MNIP, the re-binding efficiencies of imidacloprid were found to be 40.6 ± 3.1 %, 42.7 ± 3.5 % and 46.6 ± 3.9 % in methanol : water (50 : 50, v/v) (n = 3), for pH values of 4, 7 and 9, respectively. 

It was thought that, for MMIP, since the template has a basic nature (pKa 11.12), in acidic pH values, the sorption efficiency decreases due to a salt formation, and imidacloprid could not fit to the specific cavities on the surface of MMIP. In higher pH values, quantitative re-binding efficiency was observed, which is ascribed to the binding of imidacloprid to the specific sites and especially fitting the specific cavities on the surface of MMIP. For MNIP, as expected, at pH = 4, sorption efficiency decreases as explained above. Actually, no significant change was observed in the re-binding behavior of imidacloprid at higher pH values onto MNIPs, which can be attributed mainly to the non-specific binding sites on MNIP surface. Thus, it can be concluded that mainly the specific cavities are responsible for the binding of imidacloprid onto MMIP.

The addition of methanol into the aqueous phase improves the specific binding in water by reducing the non-specific interactions [45]. As stated in the literature, upon the addition of methanol, the non-specific binding sites were reduced, but the selective binding sites were found to be affected much less [46]. The re-binding of imidacloprid in water and methanol : water (50 : 50, v/v) were performed for MNIP, and the results confirmed the above information as the re-binding efficiency of imidaloprid in methanol : water (50 : 50, v/v) is lower than the re-binding efficiency in water, since the non-specific binding sites were reduced. However, for MMIP, there is a slight increase in the re-binding efficiency of imidaloprid in neutral aqueous phase (90.5 ± 2.4 %) when compared with the re-binding efficiency (96.3 ± 1.5 %) in methanol : water (50 : 50, v/v, pH~ 7). In this study, the appropriate percent of methanol in the aqueous phase might help the template for fitting the cavities possibly by decreasing the polarity of the solution. Thus, methanol : water (50 : 50, v/v) medium with a neutral pH (pH ~ 7) was chosen for the re-binding studies.

The imprinting factor (IF) can be defined as;

(1)IF=QMMIPQMNIP

where Q_MMIP_ (µmol/g) and Q_MNIP_ (µmol/g) can be expressed as the amount of imidacloprid adsorbed by MMIP and MNIP. Q_MMIP _was calculated as 0.094 µmol/g,whereas Q_MNIP_ was calculated as 0.039 µmol/g. Thus, the imprinting factor was found to be 2.41.

Both the difference in the re-binding efficiencies of imidacloprid onto MMIP and MNIP and the imprinting factor reveal the existence of the imprinted cavities on the surface of MMIP.

In the recovery studies, quantitative recovery was obtained for methanol (97.0 ± 2.5 %, n = 3), while the recovery values were below 50 % for other solvents. Thus, methanol : water (50 : 50, v/v, pH ~ 7) was used to re-bind imidacloprid onto MMIP, and methanol was used to recover the pesticide.

### 3.2. Scatchard analysis model of MNIP and MMIP

The evaluation of recognition properties of the molecularly imprinted polymers were determined by Scatchard analysis model [47]. The equation of Scatchard analysis can be expressed as

(2)QC=Qmax-QKd

where Q_max_ and Q are the apparent maximum adsorbed amount and the equilibrium adsorbed amount of imidacloprid (µmol/g), K_d_ is the dissociation constant (µmol/L) and C is the equilibrium concentration (µmol/L). Q_max_ also known as apparent maximum number of the binding sites. Scatchard analysis was applied by shaking 10 mg MNIP and MMIP with 2.0 mL various concentrations of imidacloprid solutions (0.05–0.3 µg/mL) in methanol : water (50 : 50, v/v, pH ~ 7) for one hour. 

As shown in Figure 2, Scatchard plots of MMIP involves two linear parts that two different binding sites were figured out for the rebinding of imidacloprid, whereas a single linear region for MNIP indicates one type of binding site on the polymeric structure [48]. Dissociation constants (K_d_) and maximum number of the binding sites (Q_max_) for the polymers were determined by using the linear regression equations.

Q_max_ values for two different binding sites of MMIP were found to be 0.063 and 0.151 µmol/g, whereas K_d_ values were calculated as 8.65 and 151.5 µmol/L. For MNIP, Q_max _and K_d_ values were calculated as 0.083 µmol/g and 416.7 µmol/L, respectively. It was verified that importing the specific cavities of imidacloprid provided an increasement in the capacity of the imprinted polymer.

### 3.3. Optimization of re-binding and recovery times on imidacloprid detection

For investigating the optimum time for re-binding, 100 mg MMIP was shaken with 5.0 mL, 0.5 µg/mL imidacloprid solution for different contact times (0–45 min) at room temperature. Upon sorption, magnet was used for separation of polymer, and the solution was injected to LC/Q-TOF/MS.

For determination of optimum time for recovery, first the re-binding procedure was applied, and, after sorption, MMIP was separated. A total of 5.0 mL methanol was mixed with MMIP. The mixture was stirred for different contact times (0–45 min) at room temperature for recovering the pesticide.

According to Figure 3, optimum re-binding and recovery times were both determined as 30 min. 

### 3.4. Selectivity of MMIP

The selectivity of the MMIP was studied using tebuconazole, acetamiprid and carbendazim, which were the similar structures and mostly used pesticides in fruit samples. 1.0 mL of 0.1 μg/mL imidacloprid solution individually and as a binary mixture of the studied compounds were shaken with 10 mg MMIP or MNIP. The selectivity of MMIP was determined using the following equations:

(3)Kd=C0-CeCexVm

(4)k=Kd(imidacloprid)Kd(competitor)

(5)k'=kMMIPkMNIP

where K_d_ is the distribution coefficient (L/g), C_0_ is the initial concentration of the compound (μg/mL), C_e_ is the final concentration after sorption (μg/mL), V is the sample volume (mL), m is the amount of polymer (mg), k is the selectivity coefficient and k’ is the relative selectivity coefficient. Selectivity parameters were shown in Table 2. Relative selectivity coefficient indicated that the imprinted binding sites on MMIP led to higher binding affinity to imidacloprid.

**Table 2:** The selectivity of MMIP.PesticideMMIPMNIPImidaclopridAcetamipridTebuconazoleCarbendazimKd (L/g)* 0.510.300.070.12k**-1.717.714.20Kd

Pesticide	MMIP		MNIP		
Imidacloprid Acetamiprid Tebuconazole Carbendazim	Kd (L/g)* 0.51 0.30 0.07 0.12	k** - 1.71 7.71 4.20	Kd (L/g) 0.16 0.37 0.089 0.33	k - 0.44 1.82 0.5	k’ *** - 3.89 4.23 8.36

### 3.5. Effect of adsorbent dose on determination of imidacloprid

The effect of adsorbent dose on the re-binding efficiency of trace levels of imidacloprid was investigated. Figure S4 shows that a minimum of 20.0 g/L MMIP is suitable for the quantitative re-binding of imidacloprid and further increase in dose did not affect the re-binding efficiency. 

### 3.6. Reusability of MMIP

Reusability is one of the most important properties of an imprinted polymer. Ten consecutive rebinding-recovery cycles were applied to same MMIP for investigating the reusability of the polymer. Re-binding efficiencies and recovery were obtained as 94.5 ± 2.0 % and 94.4 ± 3.0 % (n = 10), respectively. Thus, the same imprinted polymer can be used at least ten cycles for analysis of imidacloprid.

### 3.7. Interference effects

The interference effects of widely used pesticides, which are carbendazim, tebuconazole, pirimicarb, thiabendazole, carbofuran, thifensulfuron-methyl, chlorothalonil, triclosan and thiram on the determination of imidacloprid by MMIP-LC/Q-TOF/MS were investigated by shaking 100 mg MMIP with 10.0 μg/L imidacloprid solution together with different concentrations of pesticides. The interference effects of these pesticides were investigated, since they are being used in fruit samples and they may be applied together with imidacloprid to the fruits. Supernatant was injected to high resolution MS system following the re-binding and recovery procedures. Quantitative recovery values, 94.0%–98.0%, were obtained for imidacloprid in the presence of 100 fold-ratio of the pesticides owing to the mass resolving power of the MS system and the selectivity of the MMIP.

### 3.8. Analytical performance of the method

The method validation was done according to ICH guidelines by investigating various parameters [49]. Sensitivity of the method was described by the limit of detection (LOD) and limit of quantification (LOQ). LOD and LOQ were defined and calculated as three and ten times the ratio of the signal to noise, respectively. LOD and LOQ were determined for both re-binding and recovery procedures. For the precision of the method, relative standard deviations (RSDs) of intra-day and inter-day precisions of recovery studies at three different concentration levels of imidacloprid with 25.0, 100.0 and 250.0 μg/L were measured. The intra-day precision was evaluated by five repeated injections of samples on the same day, and the inter-day precision was evaluated by analyzing the sample once a day for five consecutive days.

The external calibration was carried out in order to obtain the linear range of the calibration graphs, since imidacloprid is an identified compound in the instrument library. Calculation of the peak areas were used for the quantitative analysis of imidacloprid. The calibration graphs obtained in re-binding and recovery studies are both linear between 10.0 – 500.0 µg/L as shown in Figure S5. LOD and LOQ were calculated as 2.6 µg/L and 8.6 µg/L for tap water, and LOD and LOQ were calculated as 3.0 µg/L and 10.1 µg/L for apple samples, respectively. RSD values of intra-day and inter-day studies were found to be in the range of 0.8%–1.2 % and 1.2%–1.6 %, respectively.

The analytical performance parameters of the developed method were compared with that of the published methods and briefly summarized in Table 3. The linear range, the LOD and RSDs of the developed method were comparable to other methods, and the method seemed to be suitable for the preconcentration and determination of trace levels of imidacloprid in real samples.

**Table 3:** Comparison of the proposed method with published methods.

Linear range	LOD	Method	Sample	Reference
5.0 – 200.0 µg/L	2.0 µg/kg	QuEChERS- HPLC-MS/MS	pistachio	[12]
50.0 – 1000.0 µg/L	48.0 µg/L	MMIP-UPLC-MS/MS	apple	[42]
5.0 x 10-7 – 1.0 x 10-4 M	12.0 µg/L	MIP/FcHT/AuNP	Apple, pear, grape,peach and tangerine	[50]
1.0 ×10−6 –1.0 × 10−4 M	208.1 µg/L	AgNDs/GNs/GCE	cucumber	[51]
7.5 × 10−7– 7.0 × 10−5 M	102.3 µg/L	imprinted PoPD-RGO sensor	pear	[52]
3.0 × 10−8 – 12.0 10−6 M	2.3 µg/L	GCE-GQD-IL-MWCNT-PANI	apple, cucumber and tomato	[53]
10.0 – 500.0 µg/L	2.6 µg/L and 3.0 µg/L	MMIP-LC/Q-TOF/MS	tap water and apple	Proposed method

### 3.9. Analytical application

The developed method was applied to both tap water and apple samples. In order to apply the method to tap water samples, in the first step, tap water was filtered through polytetrafluoroethylene membrane. Then, 5.0 mL of methanol was mixed with 5.0 mL tap water after adjusting pH value to 7 in order to obtain the re-binding medium, which was determined as methanol : water (50 : 50, v/v). A total of 10 mL of the prepared solution was shaken with 200 mg MMIP for 30 min for re-binding of imidacloprid. After sorption, MMIP was separated and stirred with 2.0 mL fresh methanol for 30 min in order to recover imidacloprid. Preconcentration factor of the analysis was determined as 2.5, since the original sample volume was 5.0 mL and final volume of the solvent was 2.0 mL.

For the sample preparation of apple samples, fruits were chopped and homogenized using blender. 15.0 mL of ultrapure water added onto 5.0 grams of sample, and the suspension was sonicated for 15 min. The suspension was filtered, and the solution was diluted to final volume of 25.0 mL with ultrapure water (final pH 7). Then, 25.0 mL methanol was added to solution in order to obtain the re-binding medium. Contact time was determined as 30 minfor shaking 25.0 mL of the solution with 500.0 mg MMIP for re-binding of imidacloprid. Upon the re-binding of pesticide, MMIP and supernatant was separated. A total of 5.0 mL fresh methanol was added onto polymer, and contact time was chosen as 30 min for recovery of imidacloprid. Preconcentration factor of the analysis was determined as 2.5, since the original sample volume was 12.5 mL and final volume of the solvent was 5.0 mL.

Since imidacloprid was not detected both in the blank tap water and apple samples, spike addition method was employed to the original samples with three different concentrations for three parallel analyses. Table 4 shows the results of the analytical application of the method. The quantitative recovery values of the samples were acquired in 92.0%–99.0% range. The accuracy and precision of the proposed method is quite good in terms of applicability.

**Table 4:** Analytical application of the proposed method.

Sample	Added (µg/L)*	Found (µg/L)*	Total recovery of added imidacloprid to the original sample (%)
Tap water	-25.050.0100.0	<LOD**23.9 ± 0.548.1 ± 0.899.2 ± 2.4	-95.9 ± 2.096.1 ± 1.699.2 ± 2.4
Apple	-25.050.0100.0	<LOD**23.1 ± 0.746.9 ± 1.197.2 ± 2.0	-92.4 ± 2.693.7 ± 2.297.2 ± 2.0

## 4. Conclusions 

This paper involves a method based on the magnetite-molecularly imprinted polymer and liquid chromatography-quadrupole-time of flight mass spectrometry for the determination of imidacloprid. Total preconcentration procedure can be completed within 1 h. The same MMIP can be used at least ten cycles for analysis (94.5 ± 2.0 % for re-binding and 94.4 ± 3.0 % (n = 10) for recovery) of imidacloprid. The results show that capacity of MMIP is higher capacity than MNIP and selective to imidacloprid. Selective and reusable magnetic polymer was used for the preconcentration of trace levels of imidacloprid prior to the chromatographic separation and highly sensitive mass spectrometric detection. The method was applied to real samples for the determination of imidacloprid. The quantitative recovery values of the samples were acquired in 92%–99% range. The developed method based on MMIP will be expected to present a different perspective to other studies for the analysis of trace levels of imidacloprid in fruit samples. 

Supplementary Materials

## References

[ref1] (2012). Determination of imidacloprid in rice by molecularly imprinted-matrix solid-phase dispersion with liquid chromatography tandem mass spectrometry. Journal of Chromatography B.

[ref2] (2015). Systemic insecticides (neonicotinoids and fipronil): trends, uses, mode of action and metabolites. Environmental Science and Pollution Research.

[ref3] (2010). Efficacy and uptake of soil-applied imidacloprid in the control of Asian citrus psyllid and a citrus leafminer, two foliar-feeding citrus pests. Journal of Economic Entomology.

[ref4] (2015). Assessment of imidacloprid and its metabolites in foliage of eastern hemlock multiple years following treatment for hemlock woolly adelgid, Adelges tsugae (Hemiptera: Adelgidae), in forested conditions. Journal of Economic Entomology.

[ref5] (2015). Environmental friendly method for urban wastewater monitoring of micropollutants defined in the Directive 2013/39/EU and Decision 2015/495/EU. Journal of Chromatography A.

[ref6] (2014). How a complete pesticide screening changes the assessment of surface water quality. Environmental Science & Technology.

[ref7] (2005). Two fatal intoxication cases with imidacloprid: LC/MS analysis. Forensic Science International.

[ref8] (2008). Fatal intoxication with imidacloprid insecticide. American Journal of Emergency Medicine.

[ref9] (2005). Regulation (EC) No.

[ref10] (2007). Analysis of pesticide residues using the Quick Easy Cheap Effective Rugged and Safe (QuEChERS) pesticide multiresidue method in combination with gas and liquid chromatography and tandem mass spectrometric detection. Analytical and Bioanalytical Chemistry.

[ref11] (2013). Assessment of DFG-S19 method for the determination of common endocrine disruptor pesticides in wine samples with an estimation of the uncertainty of the analytical results. Food Chemistry.

[ref12] (2018). Determination of acetamiprid, imidacloprid, and spirotetramat and their relevant metabolites in pistachio using modified QuEChERS combined with liquid chromatography-tandem mass spectrometry. Food Chemistry.

[ref13] (Water 2014). Preconcentration and Determination of Endocrine Disruptor Pesticides in Well Water by Solidified Floating Organic Drop Microextraction.

[ref14] (2008). Determination of neonicotinoid insecticides residues in bovine milk samples by solid-phase extraction clean-up and liquid chromatography with diode-array detection. Journal of Chromatography A.

[ref15] (2011). Determination of neonicotinoid insecticides residues in bovine tissues by pressurized solvent extraction and liquid chromatography-tandem mass spectrometry. Journal of Chromatography B.

[ref16] (2017). Selective solid-phase extraction using a molecularly imprinted polymer for the analysis of patulin in apple-based foods. Journal of Separation Science.

[ref17] (2016). Carbon nanotubes@silicon dioxide nanohybrids coating for solid-phase microextraction of organophosphorus pesticides followed by gas chromatography-corona discharge ion mobility spectrometric detection. Journal of Chromatography A.

[ref18] (2001). Solventless sample preparation procedure for organophosphorus pesticides analysis using solid phase microextraction and on-line supercritical fluid extraction/high performance liquid chromatography technique. Analytica Chimica Acta.

[ref19] (2011). Menezes Filho A et al. Microchemical Journal.

[ref20] (2007). Validation and uncertainty analysis of a multi-residue method for pesticides in grapes using ethyl acetate extraction and liquid chromatography-tandem mass spectrometry. Journal of Chromatography A.

[ref21] (2004). Rapid and simple screening analysis for residual imidacloprid in agricultural products with commercially available ELISA. Analytica Chimica Acta.

[ref22] (2019). Synthesis of a zinc-based metal-organic framework with histamine as an organic linker for the dispersive solid-phase extraction of organophosphorus pesticides in water and fruit juice samples. Journal of Chromatography A.

[ref23] (2019). Determination of residual organophosphorus thioester pesticides in agricultural products by chemical isotope-labelling liquid chromatography-tandem mass spectrometry coupled with in-syringe dispersive solid phase clean-up and in situ cleavage. Analytica Chimica Acta.

[ref24] (2017). Magnetic nanoparticles coated with poly(p-phenylenediamine-co-thiophene) as a sorbent for preconcentration of organophosphorus pesticides. Microchimica Acta.

[ref25] (2019). Extraction and preconcentration of organophosphorus pesticides from water samples and fruit juices utilizing hydroxyapatite/Fe3O4 nanocomposite. Microchemical Journal.

[ref26] (2018). In situ ionic liquid dispersive liquid-liquid microextraction coupled to gas chromatography-mass spectrometry for the determination of organophosphorus pesticides. Journal of Chromatography A.

[ref27] (2017). Development of fast, efficient and ecological method employing vortex-assisted dispersive liquid-liquid microextraction combined with fast gas chromatography-mass spectrometry for pesticide residues analysis in alcohol-content samples. Journal of Chromatography A.

[ref28] (2010). Determination of commonly used azole antifungals in various waters and sewage sludge using ultra-high performance liquid chromatography-tandem mass spectrometry. Journal of Chromatography A.

[ref29] (2020). Molecularly imprinted polymer as solid phase extraction phase for condensed tannin determination from Brazilian natural sources. Journal of Chromatography A.

[ref30] (2020). Preparation of core-shell magnetic molecularly imprinted polymers for extraction of patulin from juice samples. Journal of Chromatography A.

[ref31] (2020). Molecularly imprinted polymer thin-film as a micro-extraction adsorbent for selective determination of trace concentrations of polycyclic aromatic sulfur heterocycles in seawater. Journal of Chromatography A.

[ref32] (2016). Molecular imprinting: perspectives and applications. Chemical Society Reviews.

[ref33] (2017). Development of a lower toxic approach based on green synthesis of water-compatible molecularly imprinted nanoparticles for the extraction of hydrochlorothiazide from human urine. ACS Sustainable Chemistry & Engineering.

[ref34] (2019). Functionalized conjugated polymers for sensing and molecular imprinting applications. Progress in Polymer Science.

[ref35] (2014). Recent advances in molecularly imprinted polymers in food analysis. Journal of Applied Polymer Science.

[ref36] (2017). Application of magnetic molecularly imprinted polymer as a versatile and highly selective tool in food and environmental analysis: Recent developments and trends. TrAC Trends in Analytical Chemistry.

[ref37] (2020). Preparation of dummy molecularly imprinted polymers based on dextran-modified magnetic nanoparticles Fe3O4 for the selective detection of acrylamide in potato chips. Food Chemistry.

[ref38] (2018). Selective extraction of organophosphorous pesticides in plasma by magnetic molecularly imprinted polymers with the aid of computational design. Analytical Methods.

[ref39] (2020). Preparation of magnetic molecularly imprinted polymer for selective identification of patulin in juice. Journal of Chromatography B.

[ref40] (2018). Application of magnetic molecularly imprinted polymers for extraction of imidacloprid from eggplant and honey. Food Chemistry.

[ref41] (2018). Designing of fluorescent and magnetic imprinted polymer for rapid, selective and sensitive detection of imidacloprid via activators regenerated by the electron transfer-atom transfer radical polymerization (ARGET-ATRP) technique. Journal of Physics and Chemistry of Solids.

[ref42] (2019). Synthesis of core-shell magnetic molecularly imprinted polymer for the selective determination of imidacloprid in apple samples. Journal of Separation Science.

[ref43] (2014). Preparation of a magnetic molecularly imprinted polymer for selective recognition of rhodamine B. Applied Surface Science.

[ref44] (2014). Preparation of magnetic molecularly imprinted polymer for the extraction of melamine from milk followed by liquid chromatography-tandem mass spectrometry. Food Control.

[ref45] (2013). Molecularly imprinted polymers for clean water: analysis and purification. Industrial & Engineering Chemistry Research.

[ref46] (2004). Development of a molecularly imprinted polymer based solid-phase extraction of local anaesthetics from human plasma. Analytica Chimica Acta.

[ref47] (2018). Preparation and characterization of dummy molecularly imprinted polymers for separation and determination of farrerol from Rhododendron aganniphum using HPLC. Green Chemistry Letters and Reviews.

[ref48] (2020). Dummy molecularly imprinted microspheres prepared by Pickering emulsion polymerization for matrix solid-phase dispersion extraction of three azole fungicides from fish samples. Journal of Chromatography A.

[ref49] (2017). ICH Quality Guidelines. Wiley Online Library.

[ref50] (2020). A signal on-off ratiometric electrochemical sensor coupled with a molecular imprinted polymer for selective and stable determination of imidacloprid. Biosensors and Bioelectronics.

[ref51] (2017). Facile fabrication and characterization of silver nanodendrimers supported by graphene nanosheets: A sensor for sensitive electrochemical determination of imidacloprid. Journal of Electroanalytical Chemistry.

[ref52] (2013). Molecularly imprinted sensor based on electropolmerized poly(o-phenylenediamine) membranes at reduced graphene oxide modified electrode for imidacloprid determination. Sensors and Actuators B: Chemical.

[ref53] (Chemical 2019). Fabrication of a highly sensitive and selective modified electrode for imidacloprid determination based on designed nanocomposite graphene quantum dots/ionic liquid/multiwall carbon nanotubes/polyaniline.

