# Supplementary material for: Sensitive and selective determination of imidacloprid with magnetic molecularly imprinted polymer by using LC/Q-TOF/MS
Source: Turk J Chem. 2021 Aug 27;45(4):1237–47. doi: 10.3906/kim-2101-36 (PMC8517610; doi:10.3906/kim-2101-36)
Supplement: Supplementary file 1 — Supplementary Materials [file turkjchem-45-1237-sup001.pdf]

SUPPLEMENTAL MATERIAL

Imidacloprid was determined by using LC/Q-TOF/MS. HPLC equipped with a binary pump, an online degasser, an auto sampler and a Poroshell 120 EC - C18 column (3.0 × 50 mm, particle size 2.7 µm) was used for analysis. Composition of the mobile phase was 0.1 % formic acid in water (A) and acetonitrile (B). The gradient elution was: 0 – 0.5 min, 10 % B; 0.5 – 5 min, 70 % B; 5 – 7 min, 95 % B; 7.0 – 10 min, 95 % B; 10 – 15 min, 10 % B for equilibration of the column. Injection volume of the sample was 3.0 µL, the flow rate was 0.5 mL/min and the column was maintained at 35 °C.

MS and MS/MS analysis was performed using an Agilent 6550 iFunnel high resolution Accurate Mass Q-TOF / MS equipped with the Agilent Dual Jet Stream electrospray ionization (Dual AJS ESI) interface operating in positive ion in the following cases: drying gas flow, 14.0 L / min; nebulizer pressure, 35 psi; gas drying temperature, 290 °C; sheath gas temperature, 400 °C; sheath gas flow, nitrogen 12 L / min. The scanning range m / z was selected from 50 to 1000. Pesticides Accurate Mass Personal Compound Database Library (Pesticides_AM_PCDL) library was used for the quantification of imidacloprid.

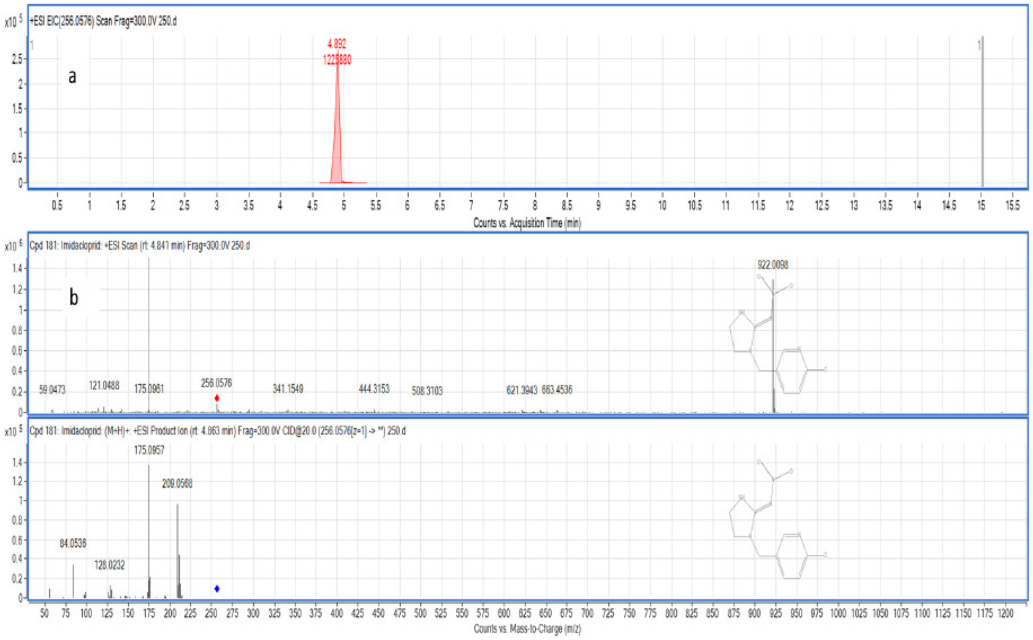

Figure S1. LC/Q-TOF/MS extract ion chromatogram (EIC) and Pesticides Accurate Mass Personal Compound Database Library (Pesticides_AM_PCDL) identification of imidacloprid.

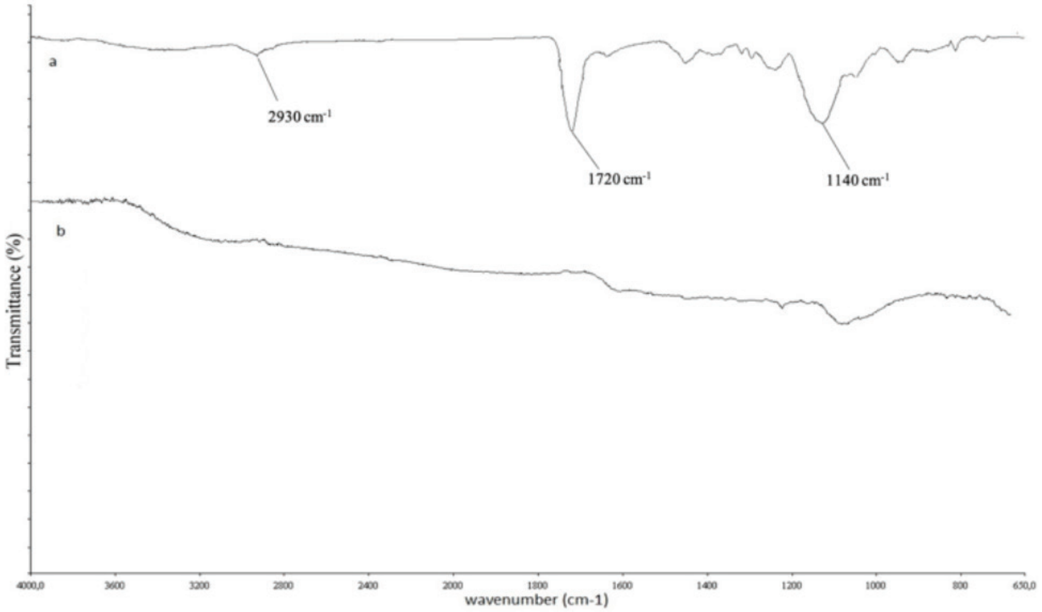

Figure S2. FTIR spectra of a) MMIP and b) magnetite particles.

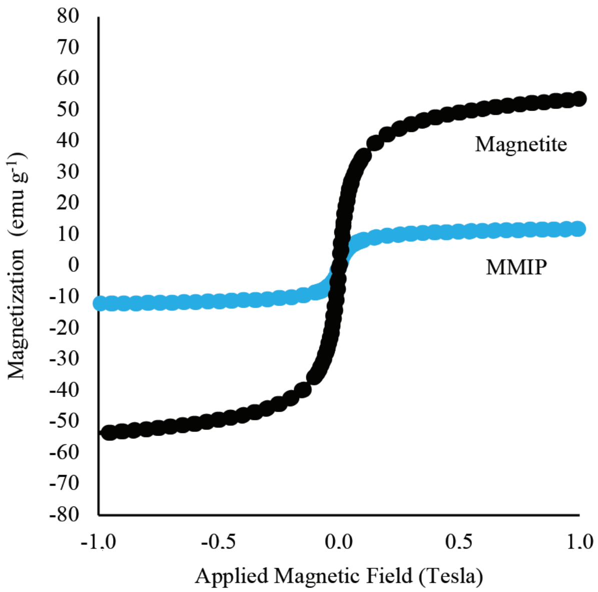

Figure S3. VSM analysis of magnetic particles and MMIP.

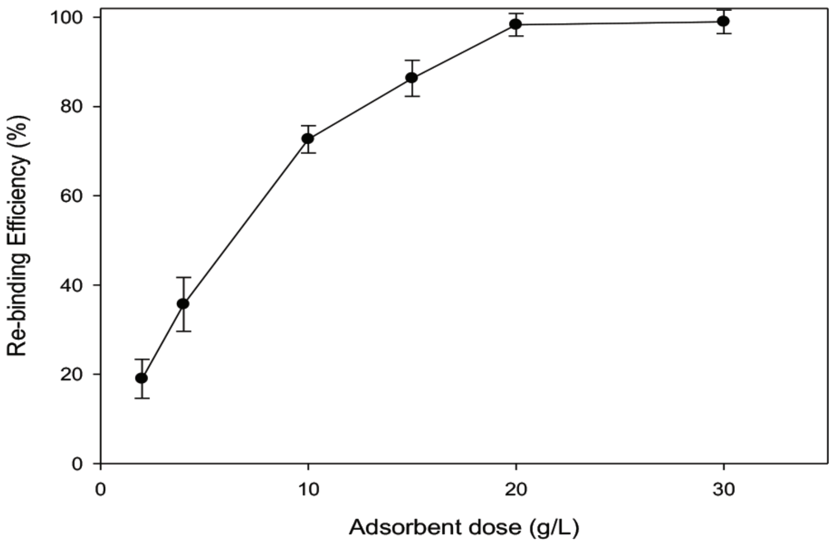

Figure S4. Effect of adsorbent dose.

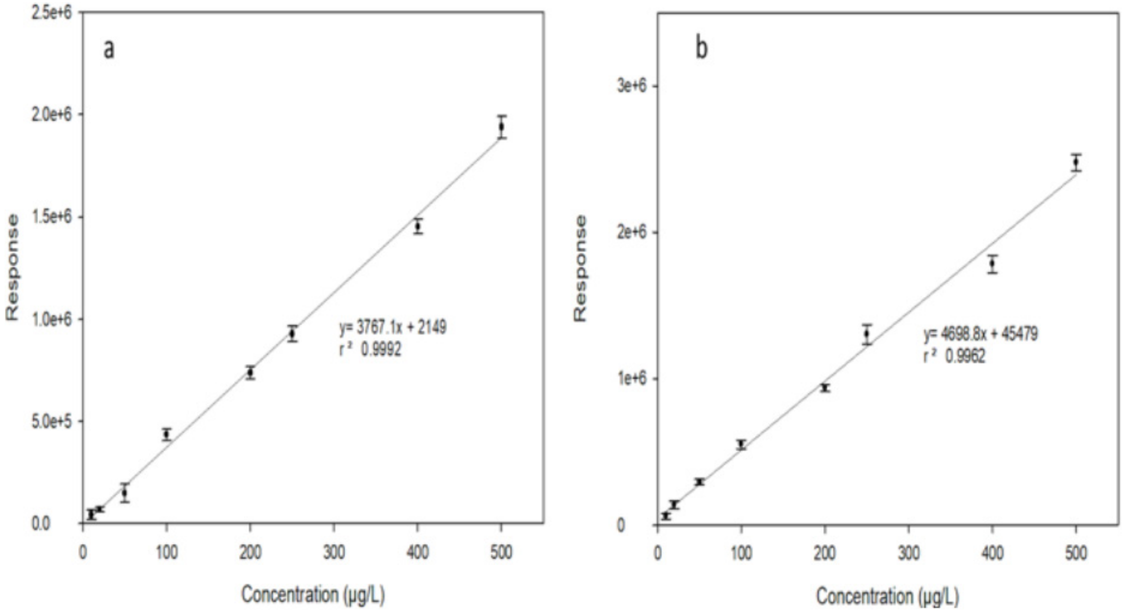

Figure S5. Calibration graph for a) re-binding and b) recovery.
